# Gender transformative innovation: Women's inclusion in livestock vaccine systems in northern Ghana

**DOI:** 10.1016/j.agsy.2024.104023

**Published:** 2024-08

**Authors:** Nelly Njiru, Alessandra Galiè, Immaculate Omondi, Dalmas Omia, Agnes Loriba, Peter Awin

**Affiliations:** a International Livestock Research Institute (ILRI), Nairobi, Kenya; b Institute of Anthropology, Gender and African Studies, University of Nairobi, Kenya; c CARE International, Tamale, Ghana; d Cowtribe, Tamale, Ghana

**Keywords:** Gender-equitable Agri-food systems, Livestock, Gender transformative approaches, Gender accommodative approaches, Gender norms, Livestock vaccines

## Abstract

**CONTEXT:**

Owning livestock can support women's empowerment and progress toward gender-equitable agri-food systems. Gender inequality, however, can reduce women's ownership of livestock and access to animal vaccines. Gender accommodative approaches (GAAs), and more recently gender transformative approaches (GTAs), are increasingly implemented in development interventions. However, their impact on women's empowerment has not been systematically tested. Here, we describe the ‘Women Rear Project’, implemented in northern Ghana between 2019 and 2023, and assess the impact of GTAs and GAAs on women's empowerment, ownership of livestock, and access to animal vaccines.

**OBJECTIVE:**

We sought to systematically assess changes conferred by GAAs and GTAs on gender norms and women's empowerment and access to animal vaccines.

**METHODS:**

Using a mixed-methods approach, in 2021 and 2023 we collected quantitative and qualitative data regarding women's empowerment, gender norms, and access to animal vaccines. Qualitative data regarding women's and men's conceptualizations of gender norms and women's empowerment were obtained via focus group discussions and key informant interviews. Quantitative survey data were obtained from 500 households, using the Women's Empowerment in Livestock Index (WELI). Quantitative data on livelihood indicators were also collected, using the Rural Household Multi-Indicator Survey (RHoMIS) tool. We systematically assessed changes between baseline (2021) and endline (2023) in local conceptualizations of empowerment in the livestock sector, the effects of GTAs and GAAs on such conceptualizations, and how empowerment was experienced by women and men and the implications for women's access to animal vaccines.

**RESULTS AND CONCLUSION:**

Local conceptualizations of empowerment in 2021, among both women and men, emphasized financial independence, decision-making autonomy, self-reliance, and problem-solving skills. In 2023, this conceptualization of empowerment had expanded to include the ability to act without external restrictions; women were empowered by managing resources, running successful businesses, and making decisions in consultation with their husbands. Furthermore, women in communities where GTAs had been enacted scored significantly higher in empowerment compared with women in communities where only GAAs had been introduced. Gender norms impacted empowerment indicators, with respect among household members and autonomy in income both contributing to disempowerment. Gender norms also affected respondents' reporting. Women's access to livestock vaccines was more positive in GTA/GAA communities. We recommend efforts are made to reduce restrictive gender norms and enhance women's empowerment and access to resources such as animal vaccines.

**SIGNIFICANCE:**

We offer recommendations for pathways toward women's empowerment and healthy livestock via inclusive innovations in agri-food systems.

## Highlights

• It is critical to address restrictive gender norms to support women's access to animal vaccines.
• Gender transformative approaches are more effective than accommodative approaches to improve women's empowerment.
• Gender transformative interventions are crucial to support the empowerment and nutrition of women.
• Quantitative data that shows disempowerment trends may obscure pathways to empowerment.
• Disempowerment of women may potentially limit their ability to effectively participate in studies.

## Graphical abstract

**Figure:**
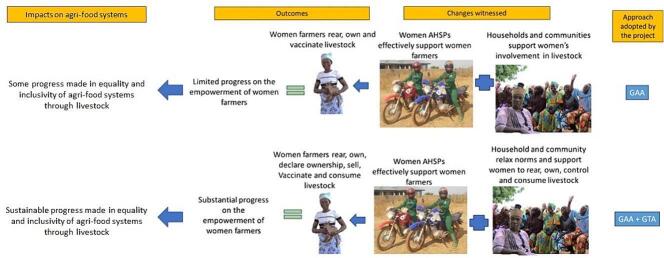

Editor: Laurens Klerkx; Guest Editor: Amy R. Beaudreault

## 1 Introduction

Ongoing efforts to transform the global food system have focused on resilience and inclusivity (FAO, 2018). Following the United Nations Food and Agriculture Organization (FAO) definition, we consider the “resilience of food systems” to be the capacity of the food system to deliver desired outcomes when exposed to stresses and shocks, in the context of economic, social, and environmental pillars (FAO, 2018). In terms of “inclusivity of food systems” we focus on gender equality and women's empowerment, which are at the heart of sustainable development (Collins, 2021).

Livestock is a key contributor to resilience in agri-food systems, supporting food and nutrition security both directly and indirectly. Directly, livestock contributes high-density macro- and micro-nutrients, critical for infants, children, and women of childbearing age (Alonso et al., 2019; McKune et al., 2021). Indirectly, livestock enhances crop production by providing manure, traction for tilling land and transportation, and cash through the sale of surplus livestock products (Mottet et al., 2017). Livestock also serves cultural and religious functions that are important for livelihoods in low- and middle-income countries (LMICs) (Alders et al., 2021; Schneider and Tarawali, 2021.).

Livestock especially small ruminants and poultry are also a key contributor to inclusivity in agri-food systems, by providing entry-points to progress toward gender equality through women's empowerment. These livestock are easily accessed and controlled by women compared with other assets, providing income that women can control and constituting a “mobile bank” that women can use to accumulate wealth or liquefy to deal with financial shocks (Galiè et al., 2019; Omondi et al., 2022). However, the value of livestock for both agri-food system resilience and gender-based inclusivity can be limited by animal diseases (Acosta et al., 2023; Hennessy and Marsh, 2021; Rushton et al., 2018). In Ghana, small-scale poultry, and goat production (characterized by low inputs and outputs) are the most prevalent livestock systems and play important roles in the livelihoods of the women farmers who are often in charge of them (Alders et al., 2021; FAO, 2012). However, their productivity potential can be reduced by diseases. Newcastle disease (ND) and peste des petits ruminants (PPR) are the primary causes of death in chickens and goats, respectively, with devastating effects on women's empowerment and livelihoods.

Vaccines are essential for preventing and controlling animal diseases (Nuvey et al., 2023). Vaccines against ND and PPR are available in Ghana, but women who keep chickens or goats rarely access them (Omondi et al., 2022)  because of discriminatory gender norms (Acosta et al., 2022; Mutua et al., 2019; Simiyu et al., 2022). We conceptualize gender norms to be the informal rules that govern the conduct of individuals based on their gender and on local conceptions of masculinity and femininity. Gender norms entrench the way things are done, how they are done, and by whom, in a manner that seems natural and unchangeable. Gender norms often reproduce and increase gender-based discrimination, e.g., by making it socially unacceptable for women to control the household income. In the case of livestock-keeping in northern Ghana, gender norms normalize the fact that only men access animal health services and products.

“Gender transformative approaches” (GTAs) is an umbrella term that refers to research and development tools that consider gender norms to be the root causes of gender-based disadvantage. GTAs aim to intentionally address restrictive gender norms at various levels of society, to address imbalances in gender-based power dynamics and relations, rigid and restrictive gender roles, and harmful practices (Cislaghi et al., 2019; FAO, 2023). Conversely, “gender accommodative approaches” (GAAs) work around existing gender-based constraints without intentionally addressing the associated gender norms i.e., the underlying structural barriers that create the constraints (Cole et al., 2020). In recent years, GTAs have increasingly been adopted to address structural factors that lead to gender-based inequalities in various development domains (Dunkle et al., 2020; Sharma et al., 2020; Udenigwe et al., 2023; Wong et al., 2019), including aquaculture/agriculture (Cole et al., 2020; Timu and Kramer, 2023; Wong et al., 2019); sexual and reproductive health, including prevention of gender-based violence (Casey et al., 2018; Dunkle et al., 2020; Ruane-Mcateer et al., 2020; Sharma et al., 2020); and natural resource management (Huyer et al., 2019). Despite the potential for GTAs to transform gender norms, their impact on women's empowerment and in combination with GAAs has not been systematically investigated, particularly in the livestock sector.

Here, we describe a study in which we systematically analyzed the impact of GTAs and GAAs on the empowerment of women livestock farmers. We also explored how gender norms changed in association with these two approaches and how women's access to animal vaccines changed. This work formed part of the “Women Rear Project” (WRP),^1^ which was established in 2019 to explore how to enhance women's access to animal vaccines for ND and PPR, thereby supporting women's empowerment, by adopting GTAs and GAAs. The project was conducted in separate communities in two districts of northern Ghana, by a consortium involving three organizations: the International Livestock Research Institute (ILRI), CARE International Ghana (CARE), and Cowtribe. The work was supported by the Livestock Vaccine 10.13039/100012774Innovation Fund (LVIF). The partners had a unique mandate, with ILRI guiding the research process, CARE implementing the GTAs and GAAs, and Cowtribe promoting access to and use of vaccines, particularly among women farmers (White et al., 2014). We used data from WRP to study the impact of GTAs and GAAs on women's empowerment, and how addressing gender norms could influence women's access to animal vaccines in the context of vaccines for goats and chickens in northern Ghana. Our primary objective was to explore how GTAs and GAAs compare in terms of their impact on women's empowerment.

## 2 Methods

We aimed to systematically assess changes in women's empowerment, gender norms, and women's access to ND and PPR vaccines at the community level following the introduction of GTAs or GAAs. A combination of GTA and GAA interventions was implemented in one set of communities, while another set of communities received GAA interventions only. We used quantitative and qualitative approaches to assess changes in women's empowerment in 2021 (before the interventions) and 2023 (following the interventions).

### 2.1 GTA and GAA models implemented by WRP

WRP was a three-year project implemented between 2019 and 2023, as a research for development project. WRP was designed to test two innovative approaches for vaccine delivery, one gender accommodative and one gender transformative, by adapting CARE International's^2^ gender transformative Farmer Field and Business School approach to facilitating women's sustained involvement in livestock vaccination. Both approaches aimed to address 1) the practical barriers faced by all farmers regardless of gender, such as the availability of animal vaccines in the area; 2) the practical barriers faced by women only such as access to available animal vaccines; and 3) the gender norms driving the barriers faced by women only such as gender norms around decision-making and women's mobility. Implemented in the Bawku West and Pusiga districts of northern Ghana, the project anchored its activities in villages/communities with active village savings and loans associations (VSLAs), as they represent a mechanism likely to support women's access to vaccines (affordability and reach). In each of the two districts, five communities were randomly selected for a GAA intervention only and five communities for a GTA intervention in combination with the GAA intervention, from a list of communities with active VSLAs (i.e., a randomized controlled trial (RCT) design), resulting in a total of 20 selected communities. Randomization by group (such as community) may be less efficient statistically than randomization by individual but was chosen based on the feasibility of delivery of the intervention and to avoid contamination between individuals allocated to competing interventions. Furthermore, a community-randomized trial, coupled with a rigorous evaluation of the intervention, can be used effectively to investigate a multichannel community-based approach to lifestyle modification, thus providing generalizability (Green, 1997). As summarized in Table 1, the GAAs mostly focused on training women farmers in animal management with an emphasis on animal health, while the GTAs built on the GAA interventions by engaging communities to explore the value (or otherwise) of local restrictive gender norms.

**Table 1:** The GTA and GAA interventions implemented.

Intervention type	GTA interventions	GAA interventions
Social	Raising awareness of the unbalanced workload between women and men and women's limited access to vaccines Recruitment of women animal-health service providers Recruitment and training of lead farmers at the village level Revamping of existing and formation of new village savings and loans associations (VSLAs) Gender training for government animal-health service providers Animal husbandry trainings for project participants Reflective community dialogues Engagement of men in dialogues about the local norms around women and livestock Engagement of men as gender champions Gender trainings Posters showing women involved in livestock farming distributed among households Radio programs sensitizing rural communities to the importance of women's involvement in livestock and livestock vaccination	• Raising awareness of the unbalanced workload between women and men and women's limited access to vaccines • Recruitment of women animal-health service providers • Recruitment and training of lead farmers at the village level • Revamping of existing and formation of new VSLAs • Gender training for government animal-health service providers • Animal husbandry trainings for project participants
Technological	● Gender-responsive digital tools ● Use of drones to deliver vaccines ● Improved vaccine delivery chain	• Gender-responsive digital tools • Use of drones to deliver vaccines • Improved vaccine delivery chain

As part of the accommodative approaches Cowtribe hired two women veterinarians to provide women farmers with animal health services. Recruiting women veterinarians helped navigate the gender norms prevalent at the study sites, where women farmers were restricted from interacting with the male veterinarians who dominated the provision of veterinary services, thereby making such services de facto inaccessible for women (Omondi et al., 2022). Women farmers were registered by Cowtribe, through a gender-responsive digital platform, which helped to map and profile the women farmers in the study communities. Drones were used to promptly deliver animal vaccines to the recruited women animal-health service providers in a short timeframe (compared with delivery by road), which helped to both maintain the cold chain and reduce costs. The GTAs built on the GAA interventions. They also engaged communities in dialogues and reflections on gender-discriminatory norms and their effects on women's engagement in livestock enterprises and ultimately on the wellbeing of households. Male and female village leaders in the study communities were involved in these dialogues. Some men and women from each community were engaged as gender champions to sensitize their communities regarding “positive masculinity” and the benefits of supporting changes to restrictive gender norms. Table 1 includes a full list of GTAs and GAAs.

### 2.2 Study area

**Fig. 1:**
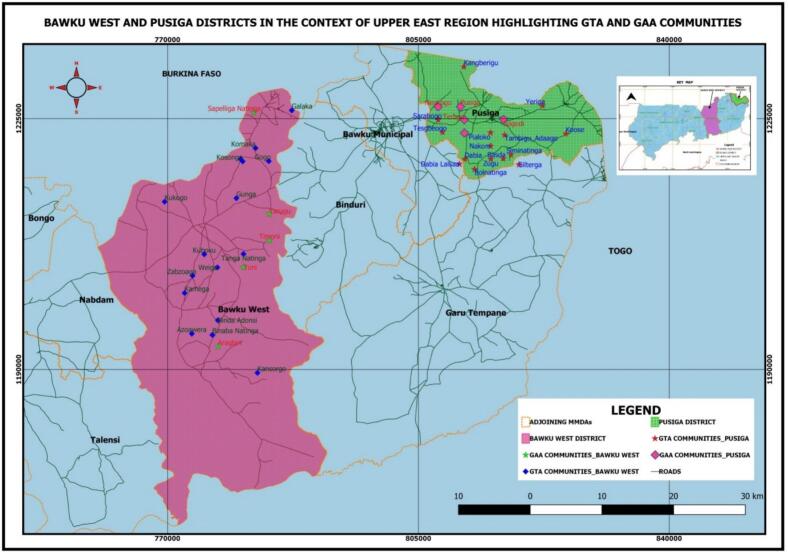
Map showing the study sites in Pusiga and Bawku West in northern Ghana.

This study was implemented in two districts in northern Ghana: Bawku West and Pusiga districts in east region (Fig. 1). These districts were selected because of the importance of small ruminants and poultry for the livelihoods of their mainly livestock-keeping population. Challenges these livestock keepers face include the high rate of livestock mortality from priority diseases whose control is only possible through vaccination and the limited access to vaccines, especially for women livestock-keepers. Approximately 49% of households are severely food insecure and mostly rely on chickens and goats for their livelihood (Kolog et al., 2023) . Kusasis are the predominant ethnic group in the district, followed by Frafras, Kasenas, Mamprusis, Moshies, Busangas, and Fulanis. There are also Ewes, who are settler fishers along the White Volta at Zongoyiri (Ghana Statistical Service, 2014).

Similarly, in Pusiga district, Kusasis are the dominant ethic group; other groups include Bissas, Moshies, and Mamprusis (Ghana Statistical Service, 2014). More than half of the population in these districts depends on livestock for food and income. Rearing goats and chickens are important for the livelihoods of both women and men (Nuvey et al., 2023). Although women play important roles in crop farming and livestock management, gender-based barriers limit their access to agricultural supplies, particularly fertilizer and animal vaccines. Women also own less livestock and mechanical equipment and have fewer years of education compared with men (FAO, 2012; 2010). Additionally, the few veterinary officers are under-resourced, with most, if not all, practicing animal-health workers being men. According to Omondi et al. (2022) “the vaccine delivery system [in Ghana] results in vaccines being more accessible to male farmers than to female farmers, largely due to the current gender norms and inequalities (e.g., women's mobility constraints, financial constraints, and social norms that govern male–female interactions)”. Other norms prevalent in the study area included assigning ultimate power in a household to men; norms regulating livestock ownership; norms that bestow or deny livestock-use rights to women or men; norms regulating livestock utilization in terms of marketing and consumption; and norms regulating access to animal health services (Njiru et al., submitted). Christianity is dominant in Bawku West, which is relatively urban, while Islam is dominant in Pusiga, which is relatively rural and located approximately one hour's drive from Bawku West.

### 2.3 Research design, data collection, and data analysis

Quantitative and qualitative data were collected from WRP study sites. An overview of our research questions can be found in Supplementary Table 1, along with the tools used and the numbers of participants interviewed during focus group discussions (FGDs), key informant interviews (KIIs), and the household surveys.

#### 2.3.1 Qualitative data collection

Between April 2021 and September 2023, we conducted a series of FGDs and KIIs with various relevant stakeholders (see Supplementary Table S1 for details). The FGDs and KIIs were conducted in local languages. They were audio-recorded with the participants' permission. The qualitative data we collected comprised information relating to local conceptualizations of women's empowerment among women and men, in the context of the livestock sector; information relating to gender norms; and the effect of the GTA and GAA interventions on women's empowerment and access to animal vaccines. In April and June 2021, we used the qualitative WELI tool to collect qualitative data on empowerment, from 12 FGDs (8 women only and 4 men only). In February and March 2023, we gathered empowerment data from 16 FGDs (8 women only and 8 men only). We also gathered data from 2 FGDs (with 16 women participants only) and a KII with 1 man. We were seeking to assess whether the local conceptualization of empowerment had changed following the implementation of the GTA and GAA interventions, and the way empowerment was experienced by women and men, in the study communities. In September 2023, additional FGDs and KIIs were conducted to validate our initial findings around women's empowerment and gender norms, through 6 FGDs (4 women only and 2 men only) and 5 KIIs (one woman and four men).

#### 2.3.2 Qualitative data processing and analysis

The audio recordings from FGDs and KIIs were transcribed, translated into English, and written down verbatim by experienced research assistants. The transcripts were checked for clarity and completeness. We used NVivo 11 software to organize, code, and disaggregate the textual material for qualitative analysis. The data were then deductively analyzed based on the themes that emerged and guided by the core gender norms that had been identified at baseline in 2021. To understand the ways in which changes in gender norms can vary with context and interact with other aspects of identity, factors such as participants' age and the community (GTA/GAA) they were from were analyzed.

#### 2.3.3 Quantitative research design

We used a pre−/post-treatment longitudinal household survey design. This involved collecting data relating to empowerment from the same households sampled in 2021 and 2023, i.e., a “before-and-after” evaluation approach. This was performed in both GAA and GTA/GAA communities. We used the Women's Empowerment in Livestock Index (WELI) tool (Galiè et al., 2019) to investigate women's empowerment. We collected additional quantitative data regarding livelihood indicators from the same survey respondents, using the Rural Household Multi-Indicator Survey (RHoMIS) tool. We also gathered data relating to perceptions about gender norms, using an additional norm module that was added to the WELI tool in 2023.

#### 2.3.4 Quantitative sampling and data collection

The minimum required sample size was arrived at based on power calculations detailed in Omondi et al. (2022). The study respondents were randomly selected from the project participants using an RCT design approach, as follows. We used a multi-stage sampling process involving a random selection of 10 communities with active VSLAs in each of the two study districts (five communities with a GAA intervention only and five communities with a combined GTA/GAA intervention). Then, we randomly selected 500 women and 100 men using a sampling frame generated from the communities at baseline (Supplementary Table S2) and followed up with the same households at endline. To assess changes in the adoption of the ND (strain I2) and PPR vaccines, we used data gathered by Cowtribe through their Zhulia platform and disaggregated by GAA and GTA/GAA interventions. We compared the total number of animals vaccinated using these vaccines in 2021 and 2022.

##### 2.3.4.1 Quantitative data processing and analysis

To assess the changes in the empowerment of women, quantitative data collected using the WELI tool were analyzed in Stata version 17 and MS Excel. The WELI index was derived as a weighted average of responses to questions that formed the 13 indicators of empowerment plus the gender parity index derived from responses of the main adult male in a household (where males were interviewed). A threshold of being adequate in at least 75% of the indicators is the standard approach used to assess whether a woman is empowered. We used means and frequencies to assess differences in empowerment over time (in 2021 and in 2023) and between treatments (GTA/GAA and GAA) and study sites (districts). We performed standard tests of means to assess whether the observed changes were statistically significant, and we explored the specific indicators of empowerment-related norms by assessing the contribution of these indicators to disempowerment over time and by treatment and district.

We also analyzed the drivers of changes in empowerment using a binary logistic model, to determine whether the changes in restrictive gender norms, which could in turn be linked to the GTA/GAA intervention, were significant drivers of changes in empowerment. Binary logistic regression is a statistical modeling technique in which the probability of dependent categorical variables is related to independent variables that are numeric or categorical (Nurdiansah and Khikmah 2020). This is a commonly used approach in studies analyzing factors contributing to changes in poverty status (Rusnak, 2012) but it has also been used to assess determinants of empowerment (Riaz and Pervaiz, 2018). The response variable (Y) in our study was changes in empowerment; this was a categorical variable (where remaining/becoming disempowered = 0, while remaining/becoming empowered = 1. The explanatory variables we used included the livelihood indicators of income, production, poverty, nutrition, and household size, and individual characteristics such as age and education level. These variables were obtained from both the quantitative WELI and RHoMIS survey data. For these analyses, we used the non-attritted dataset, generated by interviewing the same households at baseline and endline (Table 2).

**Table 2:** Quantitative survey data.

District	Treatment group	Quantitative survey (using the WELI tool)		
		No. of men	No. of women	Total
Bawku West	Five GAA communities	25	115	140
	Five GTA/GAA communities	16	107	123
Pusiga	Five GAA communities	21	107	128
	Five GTA/GAA communities	23	124	147
**Total**		**85**	**453**	**538**

To assess the effects of GTAs and GAAs on women's access to animal vaccines, we considered data on the numbers of chicken and goats vaccinated, with the i2 poultry vaccine and the PPR goat vaccine, respectively, by Cowtribe in 2021 and 2022. We opted to monitor the number of animals vaccinated, rather than the number of women who accessed the vaccines, because in many cases there was just one woman responsible for ordering vaccines and distributing them to other women in their community. The number of women who accessed vaccines through the Cowtribe platform was therefore much smaller than the actual number of women who used the vaccines.

## 3 Results

### 3.1 Socio-demographic profile of the qualitative study participants

We conducted FGDs with 294 livestock farmers (197 female; 97 male) (Table 3); we also conducted 37 KIIs (20 women, 17 men) with different value chain actors: lead farmers, religious and community leaders, women, and men farmers with or without access to vaccines, public and private vets, and individuals who were known to deviate from gender norms (we call them ‘positive deviants’) and their spouses. In Bawku West, 64% of women and 31% of men were illiterate. Illiteracy levels were higher in Pusiga, where 71% of women and 40% of men were illiterate. The average age of women and men was 39 years, and 85% of them were married, either in monogamous or polygamous households. The average household size was seven people. The majority based their livelihoods on multiple activities, mainly agriculture, with 87% (85% of men and 88% of women) engaged in farming as their primary occupation (all kept chickens and goats, an inclusion criterion for the project).

**Table 3:** Socio-demographic profile of qualitative study participants.

		Bawku West		Pusiga		Overall (%) (*n* = 294)	
		Men (%) (*n* = 32)	Women (%) (*n* = 69)	Men (%) (*n* = 60)	Women (%) (*n* = 126)	Men (%) (*n* = 97)	Women (%) (*n* = 197)
Education	College/university	16	3	5	2	13	3
	Senior secondary	19	9	13	6	14	7
	Junior secondary	9	10	28	10	21	10
	Primary	25	14	13	11	16	12
	No formal education	31	64	40	71	35	68
Marriage	Married	81	83	82	89	84	87
	Widowed	0	16	0	11	0	13
	Divorced	0	1	0	0	0	1
	Unmarried	19	0	18	0	19	0
Household size		9	8	9	6	7	9
Occupation	Farming	84	88	92	90	85	86
	Trading/other	12	7	5	10	11	12
	Student	6	0	3	0	4	0
Mean age (years)		37	38	38	41	40	38

In both districts, women commonly reared poultry, including chickens, fowl, guinea fowl, and ducks. They also reared small ruminants, such as goats and sheep, and a few reared pigs. Rearing large ruminants, such as cows, is difficult for women due to the cost and the difficulty in restraining large animals on their own. Men commonly rear larger animals, such as cattle, donkeys, and pigs. Our results revealed that 56% of women and 54% of men considered small ruminants to be the most important livestock group for supporting the livelihood of their households, followed by large ruminants. When asked which group was most important for their own livelihood, 56% of women ranked small ruminants as the most important group for their own livelihood, followed by poultry (37%); 47% of men considered small ruminants most important, followed by poultry (29%) and cattle (22%).

### 3.2 Changes in gender norms associated with GAA/GTA interventions

The data collected in 2021 revealed that both GTA/GAA and GAA communities had restrictive gender norms that affected women farmers. These included the norms that it is not acceptable for a woman farmer to own animals (i.e., to control the management of an animal and the income generated from it), declare publicly that they own an animal, interact with male veterinarians, or sell or purchase animals. It was believed that if women engaged in any of these activities, they would become arrogant and disrespectful toward their husband. It was not considered appropriate for females to eat certain animal-source foods, such as fowl meat and dogs, because, the respondents argued, men would not have enough meat to eat if women were consuming these foods. If women broke these norms, they faced disapproval and mockery from their community and hostility from their husband and other family members.

We next used the data collected in 2023 to assess whether these norms had changed, qualitatively or quantitatively. Qualitatively, in both GTA/GAA and GAA communities, the number of women engaging in livestock rearing had increased. In GAA communities, some women were able to own the animals they reared. In GTA/GAA communities, most women stated they were now able to own chickens and goats. These changes in the norm regarding women owning livestock were consistent with the quantitative results (Table 4), in which we explored how perceptions of norms had changed during the past three years, preceding the survey in 2023. Approximately 42% of women felt that this norm had changed in the past three years, with just 39% of women (44% in GAA and 35% in GTA/GAA communities) feeling that the norm still existed. We also quantitatively assessed whether the respondents complied with the norm. Non-compliance was considered to indicate relaxation of the norm. Approximately 45% women (42% in GAA and 48% in GTA/GAA communities) were non-compliant. In terms of the norm regarding selling livestock, women from GTA/GAA communities in some FGDs declared that they had started frequenting markets to buy and sell animals; such change was not seen in GAA communities, where women only sold animals through their husbands. The quantitative results suggested that approximately 55% of women (58% in GAA and 53% in GTA/GAA communities) thought the norm still existed. More than 50% of women (56% in GAA and 58% in GTA/GAA communities) complied with this norm.

**Table 4:** Percentage of women respondents who perceived the existence of, compliance with, and agreement with local gender norms, by treatment group.

Norm	Response group	Existence of the norm	Non-compliance with the norm	Agreement with the norm	Perceived change in the past 3 years
Women are not allowed to own livestock, and if they do, they are not allowed to make public declarations about animals they own	All (women) (*n* = 457)	38.73	45.08	31.95	41.58
	GAA (women) (*n* = 222)	43.5	41.5	45	42
	GTA/GAA (women) (*n* = 235)	35.02	47.86	21.78	41.25
A woman may not autonomously decide to go to the market to buy or sell livestock, but she must rely on her husband to make decisions regarding the purchase or sale of livestock	All (women) (n = 457)	55.14	42.45	52.08	22.54
	GAA (women) (n = 222)	57.5	43.5	54.5	27
	GTA/GAA (women) (n = 235)	53.31	41.63	50.19	19.07
Women and girls of a certain age or status are not allowed to eat certain livestock products	All (women) (n = 457)	19.5	44.5	28	33.7
	GAA (women) (n = 222)	26.07	44.75	21.78	36
	GTA/GAA (women) (n = 235)	34.04	42.98	31.06	31.91

We next explored changes in the norm regarding food consumption. The qualitative results suggested that women in the GTA/GAA communities, particularly younger women, could now consume animal-source foods, such as fowl meat. This was not the case in the GAA communities or, overall, for older women, who adhered to traditional norms and did not consume fowl meat. The quantitative results showed that 34% of women (36% in GAA and 32% in GTA/GAA communities) had witnessed changes in the norm around the consumption of animal-source foods in the past three years. Approximately 74% of women in GAA and 66% in GTA/GAA communities said the norm no longer existed, with approximately 45% of women in GAA communities and 43% in GTA/GAA communities no longer complying with the norm. Both women and men who participated in the FGDs and KIIs attributed such changes in norms to increased formal education and to activities organized by WRP, such as trainings on gender equity, community dialogues involving both women and men, and mobilization of community leaders and gender champions to sensitize their community about the importance of women's involvement in livestock rearing.

### 3.3 Changes in women's empowerment and its local conceptualization

In 2021, the local conceptualization of empowerment revealed a strong emphasis on four domains: financial independence, decision-making autonomy, self-reliance, and problem-solving skills. Participants generally viewed empowerment as the ability to have financial autonomy. Women considered empowerment to be when they were allowed to have their own farms and engage in businesses of their choice. The resulting financial autonomy would enable them to contribute to their family's upkeep. Men empowering their wives by including them in decision-making processes was highlighted. For instance, women were viewed as empowered if they could take care of themselves and their families without seeking external support. Similarly, the concept of empowerment was linked to autonomy and problem-solving skills. Both genders perceived empowerment to be the ability to stand on one's own, make decisions independently, and solve problems without relying on others. This self-sufficiency was considered to extend to financial matters, with individuals empowered when they can pay e.g., school and healthcare fees without external support. In 2023, the local conceptualization of empowerment involved diverse interpretations, ranging from “nyari” to “ya'a”, as expressed in Kusaal language. “Nyari” translates to “people who can say or do what pleases them without being bothered about what other people will say” and “ya'a” translates to “the power to do something”, signifying the ability to do what one desires without being restricted by external influences.

Men's empowerment was associated with financial independence and the ability to handle various responsibilities without relying on others, ownership of material resources, social status, management/leadership/decision-making power within the community and being polygamous. Women were considered empowered when they could rear animals, cultivate crops, run successful businesses, attain economic independence and independently manage their resources, gain access to education, make decisions in consultation with their husband, clean, and provide support to their husband. Changes in women's empowerment were considered positive, evident, and undeniable by all respondents, both women and men and from all backgrounds, across both GAA and GTA/GAA communities. Our quantitative results (Table 5) confirmed an overall statistically significant difference (*p* < 0.01) between the empowerment of women in 2023 compared with 2021 in both GAA and GTA/GAA communities. Women in GTA/GAA communities had significantly higher empowerment scores compared with their GAA counterparts, in both study periods. However, the mean empowerment scores for women decreased by 0.05 in 2023, in both GAA and GTA/GAA communities.

**Table 5:** Mean WELI empowerment scores, by treatment.

Period	Mean empowerment score			*t*-test
	All (*n* = 453)	GTA/GAA (*n* = 255)	GAA (*n* = 198)	
2021 (before the intervention)	0.73 (0.01)	0.75 (0.01)	0.70 (0.01)	*t* = −3.82, 451df***
2023 (after the intervention)	0.68 (0.01)	0.70 (0.01)	0.65 (0.01)	*t* = −3.65, 451df***
Change between 2021 and 2023	−0.05 (0.01)	−0.05 (0.01)	−0.05 (0.01)	*t* = −0.30, 451df

*** indicates significance at ***p < 0.01; standard deviations are indicated in parentheses; df = degrees of freedom.

In both 2021 and 2023, women's empowerment scores in GTA/GAA communities waere higher compared with these scores in GAA communities (Table 5). When we analyzed differences by district, we found that in 2021 women in GTA/GAA communities from Pusiga scored slightly higher in empowerment compared with those from Bawku West. The opposite was true for GAA communities, with women in Pusiga scoring lower than their Bawku West counterparts. In 2023, the empowerment of women in GTA/GAA communities in Bawku West did not change, while there was a small decrease in the empowerment score in GAA communities in Bawku West compared with 2021. The margin of the decrease in empowerment scores for the GAA and GTA/GAA communities in Pusiga was more pronounced than in Bawku West (−0.07 in GTA and − 0.10 in GAA communities) (Table 6).

**Table 6:** Mean WELI empowerment scores by treatment groups and districts.

Period	Bawku West				Pusiga			
	Mean empowerment values			t-test	Mean empowerment values			t-test
	All (n = 222)	GTA/GAA (*n* = 107)	GAA (*n* = 115)		All (*n* = 231)	GTA/GAA (*n* = 148)	GAA (*n* = 83)	
2021 (before intervention)	0.73 (0.01)	0.74 (0.01)	0.71 (0.01)	*t* = −1.71, 220df*	0.73 (0.01)	0.75 (0.01)	0.69 (0.01)	*t* = −3.91, 229df***
2023 (after intervention)	0.72 (0.01)	0.74 (0.01)	0.70 (0.01)	*t* = −2.38, 220df**	0.65 (0.02)	0.68 (0.01)	0.59 (0.02)	*t* = −4.32, 229df***
Difference between 2021 and 2023	−0.01 (0.01)	0.00 (0.02)	−0.02 (0.02)	*t* = −0.354, 220df	−0.08 (0.01)	−0.07 (0.01)	−0.10 (0.02)	*t* = −0.93, 229df

*, **, *** are significance levels at *p < 0.1, **p < 0.05, ***p < 0.01; standard deviations are indicated in parentheses.

To better understand these empowerment/disempowerment trends, we assessed how the WELI indicators of empowerment (associated with the selected norms – see Methods) had been affected by the change. We found that the indicators that contributed most to the disempowerment of participants in GTA/GAA intervention areas, in both districts, comparing baseline with endline, were “respect among household members” and “autonomy in income”. At baseline, these indicators were the ones that contributed most to disempowerment in GTA/GAA communities in both districts. They also contributed the most to disempowerment in GAA communities, together with “control over use of income”. At endline, in Bawku West, the indicators that contributed most to disempowerment were “respect among household members” and “input in productive decisions – livestock” in both GTA and GAA communities. In Pusiga, in GTA/GAA communities, the indicators that contributed most to disempowerment were “respect among household members” and “control over use of income”; in GAA communities, the indicators were the same except with the addition of “input in productive decisions – livestock” (Table 7).

**Table 7:** Indicators contributing to disempowerment (proportion) of women in Bawku West and Pusiga by treatment.

Indicator	Study period	Bawku West			Pusiga		
		All	GTA/GAA	GAA	All	GTA/GAA	GAA
Autonomy in income	Baseline	0.11	0.11	0.10	0.05	0.11	0.03
	Endline	0.06	0.07	0.06	0.05	0.08	0.01
Respect among household members	Baseline	0.14	0.16	0.13	0.14	0.18	0.16
	Endline	0.14	0.12	0.16	0.14	0.14	0.13
Input in productive decisions - livestock	Baseline	0.05	0.03	0.06	0.08	0.01	0.01
	Endline	0.07	0.08	0.07	0.08	0.05	0.11
Ownership of land and other assets	Baseline	0.02	0.00	0.04	0.02	0.00	0.00
	Endline	0.03	0.02	0.03	0.02	0.04	0.00
Control overuse of income	Baseline	0.10	0.09	0.11	0.11	0.06	0.04
	Endline	0.08	0.07	0.08	0.11	0.10	0.12

Across our five women's empowerment indicators, respect was the biggest contributor to disempowerment overall (across baseline and endline, districts, and GTA and GAA communities). The indicator that saw most positive change toward empowerment was “autonomy in income”, while “input in productive decisions – livestock” saw the most negative change toward empowerment overall.

We further explored the associations between empowerment and norms that could explain the observed changes in empowerment that were attributable to the GTA and GAA interventions. We used two binary logistic models (for the GAA and GTA/GAA groups) to explore drivers of changes in empowerment for two norms from the qualitative assessment that we considered most relevant to the interventions. The two norms were: 1) women are not allowed to own livestock, and if they do, they are not allowed to make public declarations about the livestock, and 2) a woman may not autonomously decide to go to the market to buy or sell livestock but must rely on her husband to make decisions regarding the purchase or sale of livestock. Of the two models, only one, the norm on sale of livestock, showed a link between women's empowerment and changes in gender norms (Table 8). Multicollinearity was tested prior to the estimation by evaluating the partial correlation coefficients between pairs of explanatory variables in the model. The variables were not correlated. From our econometric models, the likelihood ratio chi-squared tests (LR ꭓ^2^ = 23.83 and 47.70, degrees of freedom =19; *p* = 0.03 and *p* = 0.00, respectively), for the models of the norm regarding the sale of livestock were statistically significant, indicating the suitability of the models for explaining the link between women's empowerment and changes in compliance with the norm regarding selling livestock.

**Table 8:** Factors influencing changes in empowerment: Binary logistic model results.

	GTA/GAA group				GAA group			
Dependent variable: changes in empowerment (remaining/becoming disempowered vs remaining/becoming empowered)	Odds ratio	Standard error	z-value	Marginal effect	Odds ratio	Standard error	z-value	Marginal effect
Independent variables:								
Compliance with norm on selling livestock (never) (1 = never complies, 0 = otherwise)	6.41	2.48	4.79***	0.07	1.45	0.67	0.79	0.39
Household size (male adult equivalent (log)	0.20	0.12	−2.69**	−0.22	0.33	0.19	--1.90*	−0.38
Area of cultivated land (acres)	0.74	0.18	−1.23	0.02	1.11	0.16	−0.70	−0.07
Livestock holding (Tropical livestock unit (TLU) (log)	1.21	0.17	1.40	0.03	1.18	0.18	1.10	0.04
Food Insecurity Experience Scale (FIES) score (log)	0.68	0.24	−1.11	−0.15	0.48	0.24	−1.47	−0.09
Household dietary diversity score in a bad season	0.93	0.10	−0.68	−0.05	0.80	0.10	−1.88	−0.02
Total value of activities carried out, per male adult equivalent and day (log)	–	–	–	–	0.55	0.18	−1.83*	−0.00
Value of farm produce (local currency)	1.00	0.00	−1.32	−0.12	N/A	N/A	N/A	N/A
Food availability – market orientation index (log)	1.03	0.18	0.15	0.16	2.23	0.94	1.91*	0.01
Food availability – livestock orientation index	0.22	0.34	−0.98	−0.07	0.72	0.86	−0.28	−0.35
Respondent's age in years	1.00	0.01	0.13	0.00	1.00	0.00	0.43	0.00
Education_1 (1 = has formal education, 0 = otherwise)	1.52	0.73	0.87	−0.13	0.49	0.26	−1.35	0.10
Keeps goats (1 = keeps goats, 0 = otherwise)	0.37	0.28	−1.32	−0.13	0.55	0.60	−0.55	−0.24
Keeps chicken_1 (1 = keeps chickens, 0 = otherwise)	1.88	1.88	0.63	−0.37	0.21	0.34	−0.94	−0.13
_constant	22.80	44.24	1.61		346.62	829.81	0.02**	
Number of observations	179				125			
McFadden's R-squared	0.19				0.15			
−2 log likelihood	99.42				−66.83			
Likelihood ratio statistics (degrees of freedom = 19)	47.70				23.83			
*p*-value	0.00				0.03			

*, **, *** are significance levels at *p < 0.1, **p < 0.05, ***p < 0.01.

The model results (Table 8), for the GTA/GAA group showed that, of the two norms, compliance with the norm on selling livestock significantly (*p* < 0.01) influenced the outcome of empowerment over the intervention period. We used a binary variable denoting whether a respondent 1) stayed or became disempowered in 2023 compared with 2021 or 2) stayed or became empowered in 2023 compared with 2021. One factor was positively and significantly associated with the probability of becoming/remaining empowered, compliance with the norm. A woman's non-compliance with the norm that bars women from selling livestock meant that she was positively and significantly (*p* < 0.01) more likely to stay empowered/become empowered, with a probability of 7% (i.e., the odds of staying/becoming empowered among women who did not comply with the norm were 6.41-times higher than the odds among those who complied). Household size was significantly (*p* < 0.05) and negatively related to change in empowerment, implying that, as household size increased, a woman was approximately 22% more likely to become disempowered.

For the GAA group, the market-oriented food availability index had a positive and significant (*p* < 0.10) relationship with change in empowerment. Food availability (formulated from a market orientation index) was positively and significantly (p < 0.10) associated with the probability of becoming/remaining empowered. An increase in a woman's ability to access/purchase food at the market by one score (index) increased the probability of the woman becoming/remaining empowered, with a probability of 1%. The other two significant variables, household size and total value of activities, had a negative relationship with change in empowerment for GAA communities. Similar to the results seen for GTA/GAA communities, an increase in household size increased the probability of a woman in GAA communities becoming/remaining disempowered with a probability of 38%. Furthermore, if a household engaged in few household activities, then the probability of a woman becoming disempowered was significant. A woman in such a household was more likely (*p* < 0.10) to become disempowered, by a very small margin of <1%.

### 3.4 Validation of qualitative and quantitative findings

In September 2023, we conducted a new round of qualitative fieldwork to 1) validate our qualitative findings that showed an increase in empowerment and 2) explore the possible reasons behind the overall reduced trends in empowerment identified through the quantitative data. Across all interviews, what emerged was confirmation that most women had seen an unquestionable improvement in their empowerment. Women's new ability to own and rear livestock, state they owned livestock, and control the income generated (the second two mostly in GTA/GAA communities) resulted in their increased self-esteem and self-confidence. Women's self-reported reasons for this were increased knowledge and capacity to earn money; respect from husbands, who now valued their contribution to the family economy beyond simply “bearing children”, as they said; increased decision-making power, resulting from all of the above and, in some cases, expanded to managing crops; and an enhanced asset base, as the women gradually invested their new earnings in more valuable livestock and selling them when needed, e.g., to pay their own children's school fees. These changes in empowerment are particularly important in the context of polygamy, as the respondents explained that the few resources available in their household were invested by their husband in a few of his boys (with girls and other boys excluded from education); the women have no power or money to support their own children. Earning a little money through livestock rearing meant that each wife could now contribute to the education of her own boys and possibly girls.

The reasons given by all KII and FGD participants (mostly those with the most disempowered participants and separately with the most empowered participants) to explain the negative quantitative results were that most of the disempowered women in the communities were completely illiterate, had limited mobility, had very limited exposure to meetings, and may have lacked confidence when being interviewed by a stranger (the enumerators). This resulted in these women having difficulties in understanding the questions asked during the survey. Their lack of confidence meant they did not ask the enumerators for clarifications about the questions; at the same time, they tended to answer “medium” or “high” to any question about their involvement in decision-making, because they were ashamed to admit they had “little” involvement. Some participants suggested that if these women stated they had no decision-making power this could have upset their husbands who would have looked like tyrants (in the respondents' words). Some also noted that, at baseline, they had a limited understanding of the term “decision-making”, and they thought that “asking the husband for approval to leave the house” amounted to decision-making. All of this changed at the endline when, thanks to WRP, these women had been exposed to meetings and information and were consequently clearer about the questions the enumerators were asking. In the case of GTA/GAA communities, these women also stated that both exposure to information and their ability to openly declare livestock ownership increased their self-confidence when answering the survey questions. Following the project, they also had a better idea about the meaning of decision-making and where they stood in relation to it – and could therefore more “accurately” score their level of decision-making.

When discussing changes in their self-esteem, women from GTA/GAA communities in the FGDs mentioned, unsolicited, that their husbands respected them more as wives now that they could own livestock and earn from it. They explained that before, the only value a woman brought to her husband was children. Consequently, women who could not bear children were considered unhelpful (or “useless” in the respondents' words) for the family. Now that women contributed to the family economy, their value as wives went beyond just bearing children. This answer clashed with the quantitative results where the indicator “respect among household members” was the largest contributor to disempowerment overall.

### 3.5 Changes in women's access to animal vaccines

There was a 607% increase in poultry vaccinated with ND i2 vaccine and a 7% increase in goats vaccinated against PPR (Table 9). There was much greater adoption of the i2 vaccine in GTA compared with GAA communities, with increases of 1314% and 725% in GTA/GAA communities in Pusiga and Bawku West, respectively (Table 9).GTA/GAA communities Conversely, for goats vaccinated against PPR, an increase was only seen in GTA/GAA communities of Pusiga district, with fewer goats vaccinated in other communities (Table 9). Notably, the negative percentage change was higher among the GAA communities in both districts than in the GTA communities.

**Table 9:** Total number of animals vaccinated in 2021 and 2022 with the ND i2 and PPR vaccines.

Animals (poultry) vaccinated with ND i2 vaccine					Animals (goats) vaccinated with PPR vaccine		
District	Community type	2021	2022	Percentage change	2021	2022	Percentage change
Pusiga	GTA	424	5996	1314	391	712	82
	GAA	1173	4604	292	351	191	-46
Bawku West	GTA	680	5610	725	189	180	-5
	GAA	1486	10,383	599	244	97	-60
	**Total**	**3763**	**26,593**	**607**	**1103**	**1180**	**7**

All FGD and KII respondents attributed the increase in animals vaccinated to WRP interventions, particularly the recruitment of female animal-health service providers who were more accessible to women farmers compared with male veterinarians. Cowtribe staff suggested the low number of goats vaccinated in 2022 was due to delays in farmer engagement and sensitization and reduced outreach. Furthermore, the PPR vaccination period coincided with peak cropping season, so women and men were engaged in farm work and away from home, where vaccination was to take place. Additionally, some farmers had insufficient finances to support their livestock and crop farming needs. Communities that were further away from Zipline (the company that partnered with Cowtribe to deliver vaccines using drones) experienced further delays in vaccine delivery. CARE and Cowtribe staff also suggested the decline in the number of vaccinated animals in 2022 was related to heavy rains in April–May 2022, which can lead to rapid growth of lush grass that can poison/harm animals. The project was therefore advised by the regional veterinary directorate to halt the vaccination process because farmers may have erroneously attributed animal deaths due to grass poisoning to the PPR vaccine.

The FGD and KII participants also attributed the positive change in the number of animals vaccinated in part to women's empowerment interventions. The implementation of GTAs to change restrictive gender norms increased women's access to vaccines. According to FGD participants, transformative strategies that increased their access to animal vaccines were 1) women's ability to own animals and declare this, 2) women's ability to access and interact with female veterinary officers recruited by the project, 3) the ability of some women to sell their animals, which increased their income options, and 4) sensitization.

## 4 Discussion

In this study we assessed how GTAs and GAAs changed gender norms, women's empowerment, and women's access to animal vaccines. In terms of changes in gender norms, both GTAs and GAAs were instrumental in reducing the strength of restrictive norms in all communities. GAA communities mainly saw a change in two norms (women being able to rear animals and eat meat), while GTA/GAA communities saw various degrees of change in three additional norms: the prohibition of women owning animals and publicly declaring such ownership and the discouraging of women from selling livestock. Challenging the root causes of gender inequalities in livestock-based agri-food systems can benefit women, their families, and communities (Baltenweck et al., 2022; Njiru et al., 2023; Njuki and Sanginga, 2013; Quisumbing et al., 2015). We provide much needed evidence on how GTAs (in combination with GAAs) may create greater changes in restrictive gender norms.

In terms of changes in women's empowerment, our qualitative findings revealed progress in key domains of empowerment, from conceptual transformation to decision-making to asset accumulation. These changes were clearly stronger in GTA/GAA communities. GTAs in particular appeared to move the needle on empowerment because they changed women's ability to declare that they own livestock essential to control income, take decisions, and accumulate wealth. Similarly, a strong correlation was found between changes in norms and empowerment. Clearly, all steps women made in empowerment domains depended on changes to norms that preventing women from owning livestock, declaring ownership etc. This strong link between gender norms and women's empowerment – and the progress made in changing gender norms through GTAs – speaks to the importance of these approaches to support women's empowerment.

Interestingly, our quantitative results contradicted our qualitative results, indicating a process of disempowerment overall. The research team thus returned to the field, to explore with project participants and partners their views on this contradiction. This comprised our qualitative validation fieldwork, through which we found that gender norms greatly affected the capacity of most disempowered women farmers to provide accurate answers to the quantitative survey at baseline. Exposure to the project had improved such capacity at endline. According to these findings, therefore, the quantitative data showing disempowerment are only reliable in showing how disempowered women were in 2021 (revealed through the process of conscientization self-reported in 2023), rather than a process of disempowerment over the years. In other words, the very process of empowerment the most disempowered women went through resulted in high and unreliable empowerment scores at baseline and more realistic scores at endline. Three points emerge from this: 1) quantitative assessments of disempowerment can “hide” a process of empowerment, as noted by Galiè (2013). 2) Instances when qualitative and quantitative data clash are opportunities for deeper analysis of a situation rather than a “tug of war” about which methodological approach is most reliable, as discussed by Galiè et al. (2019). 3) Gender norms can greatly affect both quantitative and qualitative data because respondents' reports are naturally affected by gender norms. In a context where women cannot own livestock, community members will only report men as being “livestock keepers”. This has important implications for quantitative data, particularly because qualitative fieldwork, unlike quantitative surveys, offers an opportunity to engage respondents in discussions and reveal norms (Lecoutere et al. 2023).

Changes in women's access to vaccines were shown to be more positive in GTA/GAA communities than GAA communities. Technical issues and adverse weather were the main reasons for slow increases in vaccine access, which require separate analysis and interventions. Gender norms were found to be important mediators of women farmers' access to vaccines. These results agree with findings reported by McKune et al. (2021) and Serra et al. (2022), who found that addressing gendered barriers in the livestock sector, which often discriminate against women, can potentially increase women's access to animal vaccines.

Overall, our findings show that unless restrictive gender norms are addressed at a project's outset, the potential of a livestock animal health intervention to support women's empowerment and access to vaccines is limited. Only by making progress on the latter can the livelihoods and nutrition of women farmers and their families be strengthened. GTAs coupled with GAAs emerged as the most effective means of relaxing restrictive gender norms. This was followed by progress in various domains of women's empowerment and increased access to animal vaccines. Women owned healthier animals that they could sell to pay for school fees, buy food, and reinvest in more valuable livestock. Because GTAs address gender norms, the root causes of gender discrimination, their adoption is key for sustainable progress toward equitable and inclusive agri-food systems involving livestock.

This study had a limitation that should be mentioned. Our experience exploring the impact of GTAs versus GAAs raised a methodological consideration related to gender norms. At baseline, we conducted a small qualitative exploration of gender norms, to shed light on gender norms that existed within the study communities, and which we needed to take into account when designing GTAs and GAAs. We did not study gender norms extensively because we had only planned to assess the impact of GTAs and GAAs on women's empowerment. At endline, we explored changes in gender norms and compared GTAs, GAAs, and communities not involved in the project for the qualitative component, and we compared GTAs and GAAs for the quantitative component. At this point in time, and after observing how important conducive gender norms are to women's empowerment, we realized the importance of systematically assessing changes in gender norms, as an end in itself and as a means to create a conducive environment for women's empowerment to materialize. However, because we missed substantial qualitative and quantitative baseline data, and relied mostly on recall at endline, our assessment of the changes in gender norms brought about by GTAs and GAAs was less systematic when compared to our assessment of changes in women's empowerment. Building on this experience, we later developed a tool to systematically assess changes in gender norms, both qualitatively and quantitatively.

## 5 Conclusion

There are increasing calls to transform the agri-food system to become more sustainable and equitable. Restrictive gender norms exacerbate societal inequalities, which play out in agri-food systems and hamper food security. In the case of livestock ownership, these norms can restrict women's access to animal vaccines, thereby hindering the potential benefits they could reap by raising healthy animals. GTAs are a promising means to address restrictive gender norms. However, little is known about how such approaches compare with GAAs. Here, we systematically studied how GTAs and GAAs impacted changes in women's empowerment and their access to vaccines. GTAs were instrumental in changing restrictive gender norms, which in turn is key in supporting progress toward women's empowerment and access to animal vaccines. Technical animal-health interventions that do not create a conducive normative environment for women to access and benefit from animal vaccines are likely to have limited success in supporting food and nutrition security in a context such as northern Ghana. The livestock sector's ability to contribute to transforming agri-food systems require a strong foundation of a vaccine delivery system that works for both women and men farmers by addressing restrictive gender norms.

## Funding

This research was part of the Livestock Vaccine Innovation Fund (LVIF) which is supported by the Bill & Melinda Gates Foundation (BMGF), Global Affairs Canada (GAC), and Canada's International Develoment Research Centre. The Sustainable Animal Productivity for Livelihoods, Nutrition, and Gender Inclusion (SAPLING) also supported the writing of this article. The views expressed herein do not necessaritly represent those of IDRC or its Board of directors.

## Ethics statement

The studies involving human participants were reviewed and approved by ILRI's Institutional Ethics Committee (Ref. ILRI-IREC2019–48/1). We also received ethical clearance from the Council for Scientific and Industrial Research (CSIR) (Ref. CSIR/IRB/AL/23/VOL1–002).

## CRediT authorship contribution statement

**Nelly Njiru:** Writing – review & editing, Writing – original draft, Visualization, Validation, Software, Project administration, Methodology, Investigation, Formal analysis, Data curation, Conceptualization. **Alessandra Galiè:** Writing – review & editing, Visualization, Validation, Supervision, Project administration, Methodology, Investigation, Funding acquisition, Conceptualization. **Immaculate Omondi:** Writing – review & editing, Software, Methodology, Investigation, Formal analysis, Data curation, Conceptualization. **Dalmas Omia:** Writing – review & editing, Visualization, Supervision. **Agnes Loriba:** Writing – review & editing, Project administration, Funding acquisition, Conceptualization. **Peter Awin:** Writing – review & editing, Project administration, Funding acquisition, Conceptualization.

## Declaration of competing interest

The authors declare that the research was conducted in the absence of any commercial or financial relationships that could be construed as a potential conflict of interest.

## Data Availability

Supplementary data for this article will be provided upon request.

## Appendix A Supplementary data

Supplementary material Image 1

## Footnotes

1 https://cgspace.cgiar.org/items/05ba4480-59de-4813-b300-a040a49d75c3

2 https://www.care-international.org/our-work/where-we-work/ghana
