# Corrigendum to ‘Gender Transformative Innovation: Women's Inclusion in Livestock Vaccine Systems in Northern Ghana’ [Agricultural Systems; 219 (August 2024) 104023]

**DOI:** 10.1016/j.agsy.2026.104772

**Published:** 2026-06

**Authors:** Nelly Njiru, Alessandra Galiè, Immaculate Omondi, Dalmas Omia, Agnes Loriba, Peter Awin

**Affiliations:** a International Livestock Research Institute (ILRI), Nairobi, Kenya; b Department of Anthropology Gender and African Studies, University of Nairobi, Kenya; c CARE International, Tamale, Ghana; d Cowtribe, Tamale, Ghana

The authors regret that in the original published version of this article, the second institutional affiliation for Nelly Njiru was inadvertently omitted. This corrigendum serves to add the missing affiliation and change from the word institute to department in the second affiliation (^b^). The authors apologize for this oversight and confirm that the correction does not affect the scientific content or conclusions of the article.

The authors would like to apologize for any inconvenience caused.

