# Supplementary material for: Gender transformative innovation: Women's inclusion in livestock vaccine systems in northern Ghana
Source: Agric Syst. 2024 Aug;219:104023. doi: 10.1016/j.agsy.2024.104023 (PMC11304341; doi:10.1016/j.agsy.2024.104023)
Supplement: Supplementary file 1 — Supplementary material [file mmc1.docx]

**Supplementary** **Table S1:** Overview of the research questions, tools used, and numbers of participants

| **Research question** | **Tool used** | **Study district** | **Study community intervention** | **Number of participants** | **Year** |
| --- | --- | --- | --- | --- | --- |
| 1. What was the impact of GTAs and GAAs on the empowerment of women livestock keepers? | Qualitative Women’s Empowerment in Livestock Index (WELI) | Bawku West | GAA | 3 FGDs: 2 with women (18 participants) and 1 with men (8 participants) | 2021 |
|  |  |  |  | 4 FGDs: 2 with women (14 participants) and 2 with men (18 participants) | 2023 |
|  |  |  | GTA | 3 FGDs: 2 with women (20 participants) and 1 with men (8 participants) | 2021 |
|  |  |  |  | 4 FGDs: 2 with women (18 participants) and 2 with men (20 participants) | 2023 |
|  |  | Pusiga | GAA | 3 FGDs: 2 with women (16 participants) and 1 with men (8 participants) | 2021 |
|  |  |  |  | 4 FGDs: 2 with women (19 participants) and 2 with men (16 participants) | 2023 |
|  |  |  | GTA | 3 FGDs: 2 with women (16 Participants) and 1 with men (9 participants) | 2021 |
|  |  |  |  | 4 FGDs: 2 with women (24 participants) and 2 with men (17 participants) | 2023 |
|  | Validation of WELI results | Bawku West | Mixed GTA and GAA | FGDs: 3 (1 with women with the lowest empowerment scores, 1 with women with the highest empowerment scores, and 1 with husbands of women with the lowest and highest empowerment scores  KIIs: 2 (1 woman and 1 man), a private veterinarian and an officer from the district veterinary office | September 2023 |
|  |  | Pusiga | Mixed GTA and GAA | FGDs: 3 (1 with women with the lowest empowerment scores, 1 with women with the highest empowerment scores, and 1 with husbands of women with the lowest and highest empowerment scores  KIIs: 3 (1 woman and 2 men), being either the private veterinarian recruited by Cowtribe, veterinary officers, and an officer from the Ministry of Food and Agriculture |  |
|  | Quantitative WELI tool  Rural Household Multi-Indicator Survey (RHoMIS) tool | Bawku West | GAA | 125 women and 25 men each study year | May/June 2021 and May/June 2023 |
|  |  |  | GTA/GAA | 125 women and 25 men each study year |  |
|  |  | Pusiga | GAA | 125 women and 25 men each study year |  |
|  |  |  | GTA/GAA | 125 women and 25 men each study year |  |
| 1. How did the gender norms change following the introduction of GTAs and GAAs? | Qualitative gender norms tool | Bawku West | Mixed GAA and GTA/GAA | 1 FGD: 1 with women (8 participants)  1 KII: 1 with a man | June 2021 |
|  |  | Pusiga | Mixed GAA and GTA/GAA | 1 FGD: 1 with women (8 participants) |  |
|  | Qualitative gender norms tool | Bawku West | GAA | 5 FGDs: 3 with women (27 participants) and 2 with men (16 participants)  3 KIIs: 1 woman and 2 men | February and March 2023 |
|  |  |  | GTA/GAA | 5 FGDs: 3 with women (32 participants) and 2 with men (16 participants)  11 KIIs: 6 women and 5 men |  |
|  |  | Pusiga | GAA | 5 FGDs: 3 with women (35 participants) and 2 with men (15 participants)  5 KIIs: 3 women and 2 men |  |
|  |  |  | GTA/GAA | 5 FGDs: 3 with women (26 participants) and 2 with men (14 participants)  10 KIIs: 5 women and 5 men |  |
|  |  |  | Non-intervention | 4 FGDs: 2 with women (36 participants) and 2 with men (16 men participants) |  |
|  |  | Pusiga and Bawku West | Project implementors | 1 FGD: 1 mixed FGD (5 men and 2 women participants)  8 KIIs: 5 women and 3 men |  |
|  | Quantitative norms module in the WELI tool^ and livelihood indicators from the RHoMIS^[[1]](#footnote-1)^ tool | Bawku West | GAA | 125 women and 25 men each study year | May /June2023 |
|  |  |  | GTA/GAA | 125 women and 25 men each study year |  |
|  |  | Pusiga | GAA | 125 women and 25 men each study year |  |
|  |  |  | GTA/GAA | 125 women and 25 men each study year |  |
| 1. How did women’s access to animal vaccines change following the introduction of GTAs and GAAs | Zhulia platform, by Cowtribe | Bawku West | GAA | PPR vaccine: 398 women farmers  II2 vaccine: 398 women farmers | 2021 |
|  |  |  |  | PPR vaccine: 447 women farmers  I2 vaccine: 398 women farmers | 2022 |
|  |  |  | GTA/GAA | PPR vaccine: 433 women farmers  I2 vaccine: 433 women farmers | 2021 |
|  |  |  |  | PPR vaccine: 427 women farmers  I2 vaccine: 433 women farmers | 2022 |
|  |  | Pusiga | GAA | PPR vaccine: 474 women farmers  I2 vaccine: 474 women farmers | 2021 |
|  |  |  |  | PPR vaccine: 447 women farmers  I2 vaccine: 474 women farmers | 2022 |
|  |  |  | GTA/GAA | PPR vaccine: 451 women farmers  I2 vaccine: 451 women farmers | 2021 |
|  |  |  |  | PPR vaccine: 415 women farmers  I2 vaccine: 451 women farmers | 2022 |

**Note:** ^recall data gathered through a gender norms module integrated in WELI tool

FGD, focus group discussion; GAA, gender accommodative approach; GTA, gender transformative approach; I2, Newcastle disease strain I2 vaccine; KII, key informant interview; PPR, peste des petits ruminants

Supplementary Table S2. Sample size for the quantitative survey.

| **District** | **Number of communities** | **Treatment group** | **Quantitative survey (using the WELI tool)** | |  |
| --- | --- | --- | --- | --- | --- |
|  |  |  | **No. of men** | **No. of women** | **Total** |
| Bawku West | 5 | GAA | 25 | 125 | 150 |
|  | 5 | GTA/GAA | 25 | 125 | 150 |
| Pusiga | 5 | GAA | 25 | 125 | 150 |
|  | 5 | GTA/GAA | 25 | 125 | 150 |
| Total | | | 100 | 500 | 600 |

1. https://www.rhomis.org/ [↑](#footnote-ref-1)
